# Neck circumference is independently associated with relative systemic hypertension in young adults with sickle cell anaemia

**DOI:** 10.1186/s40885-018-0088-2

**Published:** 2018-02-16

**Authors:** Lawrence A. Olatunji, Olatunde P. Olabode, Olawale M. Akinlade, Abiola S. Babatunde, Victoria A. Olatunji, Ayodele O. Soladoye

**Affiliations:** 1 HOPE Cardiometabolic Research Team and Department of Physiology, College of Health Sciences, University of Ilorin, P.M.B 1515, Ilorin, Kwara State 240003 Nigeria; 2 Department of Medicine, Ladoke Akintola University of Technology, Ogbomoso, Oyo State Nigeria; 3 Department of Haematology and Blood Transfusion, College of Health Sciences, University of Ilorin, P.M.B 1515, Ilorin, Kwara State 240003 Nigeria; 4 Department of Ophthalmology, University of Ilorin Teaching Hospital, P.M.B 1515, Ilorin, Kwara State 240003 Nigeria

**Keywords:** Arterial blood pressure, Cardiometabolic disorder, Neck circumference, Sickle cell disease, Upper-body fat accumulation

## Abstract

**Background:**

A seemingly interesting observation in patients with sickle cell anaemia (SCA) is that they usually have lower systemic blood pressures (BP) and insulin resistance than persons in the general population in spite of chronic inflammation and vasculopathy. However, relative systemic hypertension (rHTN) has been linked to pulmonary hypertension, increased blood viscosity and renal insufficiency, which could indicate a risk of developing cardiometabolic disorder (CMD) in SCA.

We therefore hypothesized that neck circumference (NC) and CMD marker; triglyceride glucose (TyG) index would independently predict rHTN in young adults with SCA in steady state.

**Methods:**

We compared the anthropometrical, hematological, hemorheological and CMD markers between SCA patients with normal BP < 120/70 mmHg; nHTN, *n* = 65) and those with rHTN (BP ≥ 120/70 mmHg, *n* = 32).

**Results:**

Our results showed that SCA with rHTN had significantly higher body weight, waist circumference, NC, plasma viscosity, systolic and diastolic BP. Results also indicated that NC (OR: 2.98; 95% CI 1.46 to 6.10, *p* < 0.01) was a predictor of rHTN in SCA independent of gender, age, weight, waist circumference, BMI, blood viscosity, triglyceride or TyG. A receiver operating characteristic curve analysis also showed that NC was the most efficient predictor of rHTN than other CMD markers.

**Conclusion:**

The present study demonstrates that increased NC is a salient risk factors that is independently associated with rHTN in SCA. The finding therefore underscores the utility of NC in early detection and stratification of systemic hypertension, particularly in individuals with SCA.

## Background

Sickle cell disease (SCD), one of the most common inherited blood disorders worldwide, is an autosomal recessive disorder caused by point mutations in the gene that encodes the β-globin chains of hemoglobin (Hb), leading to polymerization of erythrocytes upon deoxygenation. This in turn, results in rigid, adherent erythrocytes, which get trapped in the microcirculation. Vaso-occlusive phenomena and hemolysis are the clinical hallmarks, although other clinical occurrences are also observed [1]. This group of individuals with advanced age, could develop chronic inflammation-related organ injury, such as chronic kidney disease (CKD), atherothrombotic events, pulmonary vasculopathy or hypertension [2, 3].

Individuals with sickle cell anemia (SCA) have been shown to have lower arterial BP when compared with age- and sex-matched persons without SCA [4, 5], but fail to show the age related rise in BP common in the normal population. The prevalence of systemic hypertension is also lower in SCA [6]. The findings that patients with SCA have low BP seems to counter recent pathophysiological studies which suggest that these patients develop endothelial dysfunction and arterial vasculopathy associated with reduced nitric oxide (NO) bioavailablity that is seen in the hypertensive population [7, 8]. Also, even within a range of systolic and diastolic BP that would be considered normal by conventional standards in SCA, there is an association between increased BP and cerebrovascular accident or mortality [4, 6, 9]. These reports raise the possibility that the normal range for BP in patients with SCA appears to be higher than it might have been expected, given the severity of their anemia, vasculopathy and exacerbated organ damage. This suggests the likelihood of relative systemic hypertension in these patients (rHTN) [6].

Sickle cell disease is an independent cause of pulmonary artery hypertension and patients with SCD have been reported to have increased risk to develop pulmonary hypertension and renal dysfunction than the normal control [2]. Pulmonary hypertension has been associated with reduced survival in SCD patients [6, 9]. Also elevated systemic blood pressures had been shown to contribute significantly to left ventricular diastolic dysfunction, a phenomenon which has been reported as an independent predictor of death in SCD patients [6, 9]. Systemic hypertension is a major risk factor for cardiovascular disease (CVD) estimated to account for about 9.4 million deaths in 2010 with global prevalence in adults of about 22% in 2014 [10]. Interestingly, a group of investigators has established that BP ≥ 120/70 mmHg considerably increased the risk for atherothrombotic CVD and chronic organ injury in these individuals [2]. Early recognition and prompt treatment of relative systemic hypertension in young adults with SCD may inhibit progression to pulmonary hypertension, renal dysfunction and other cardiovascular complications thereby reducing morbidity in affected individuals.

Overweight and obesity are rising to pandemic and remain major public health problems globally and they are important risk factor for cardiometabolic disorder (CMD). The prevalence of obesity varies from 10 to 40% in different populations [11, 12]. Unfortunately, the prevalence of obesity has increased in industrialized countries and also in developing ones, including Nigeria, where there is high incidence and prevalence of SCA [13]. Several recent studies have shown that upper-body subcutaneous fat appears to confer additional risk for CMD than general and central obesity, which has been reliably predicted by the neck circumference (NC). NC is strongly correlated with insulin resistance (IR) and other CMD risk factors across several ethnicities in the general population [14–17]. Moreover, there is a growing interest in the utility of a simple but reliable marker with wide applicability; the triglyceride-glucose (TyG) index, the product of the fasting blood glucose and triglyceride (TG) levels for IR [18, 19].

Since upper-body fat accumulation may be responsible for many hormonal changes playing a role in the development of cardiovascular dysfunction and HTN in the general population, we suggest that it could be the case in SCA patients too. To the best of our knowledge to date, there have been no published studies on the association between the indices of IR and upper-body visceral adiposity, using NC or TyG in young adults with SCA. In this study, we therefore tested the hypothesis that NC and TyG index would independently predict rHTN in young adults with SCA in steady state.

## Methods

### Study population

The study was carried out in the adult outpatient sickle cell clinics of the University of Ilorin Teaching Hospital (UITH), Ilorin, Kwara State, Nigeria and Ladoke Akintola University of Technology (LAUTECH) Teaching Hospital, Ogbomoso, Oyo State, Nigeria between February, 2013 and April 2016, and included 97 (M/F = 50/47) young adults who had Hb genotype SS confirmed by Hb electrophoresis. All patients were in steady-state condition at the time recruitment into the study which was defined as absence of vaso-occlusive crisis, acute chest syndrome (ACS), stroke, priapism, and absence of any signs or symptoms attributed to acute illness, at least 8 weeks before inclusion into the study. The exclusion criteria for SCA patients were presence of congenital or acquired heart disease, pregnancy, very severe anemia (hematocrit < 18%), blood transfusions in the previous 12 weeks, use of drugs that modifies blood rheology, excessive intake of alcohol (more than 16 g daily), and use of tobacco. All patients were informed about the purpose and procedures of the study, and gave their written consent. The study was conducted in accordance with the guidelines set by the Declaration of Helsinki on the protection of the rights of human subjects and was approved by the Ethical Review Committees of UITH and LAUTECH Teaching Hospital.

### Clinical parameters

Waist circumference (WC), neck circumference (NC), hip circumference (HC), height and weight were measured for all patients using a meter tape and body mass index (BMI) was calculated as BMI = weight/height^2^, Kg/m^2^, triglyceride-glucose index was estimated as the product of fasting triglyceride and glucose expressed as TyG index; Ln [TG (mg/dl)* FPG (mg/dl)/2] [18] whereas TyG-BMI; TyG*BMI, TyG-WC; TyG*WC [Er et al., 2016]. The blood pressures of subjects were measured using the auscultatory method of Korotokoff. At the clinics, patients were made to seat comfortably in a chair with the upper arm rested on a table for 3 min before measurement of the blood pressures. Using a mercury in-glass Shygmomanometer and appropriate cuff was applied to the upper arm with the lower edge of the cuff at least one inch above the antecubital fossa. The approximate systolic blood pressures were first obtained for each patient by inflating the cuff and palpation of the brachial artery. The cuff was then deflated and re-inflated to about 10 mmHg above the approximate systolic pressure value. The cuff was then deflated and the systolic and diastolic blood pressures of the patients were recorded as the phase I and phase IV Korotokoff sounds respectively. Repeated 3 blood pressure measurements were taken after 10 min interval in each patient and recorded. The blood pressure values of the subjects were then recorded as average readings of the systolic and diastolic blood pressures. The BP values < 120/70 mmHg were considered as normal (nHTN group; *n* = 65). Patients with BP values ≥ 120/70 mmHg and < 140/90 mmHg were considered as having rHTN (*n* = 29); patients with BP levels ≥ 140/90 mmHg had hypertension (HTN) (*n* = 3) [2]. Mean arterial pressure (MAP) was calculated for the two groups as the diastolic $$ \mathrm{BP}+\raisebox{1ex}{$1$}\!\left/ \!\raisebox{-1ex}{$3$}\right. $$ pulse pressure. Heart rate (HR) was also measured for each patient.

### Biological parameters

Blood samples were drawn after a 12 h overnight fasting, between 8:00 a.m. and 10:00 a.m. For hematological parameters such as Hb and hematocrit (Hct) concentrations; red blood cell (RBC), white blood cell (WBC) and platelet (PLT) counts were determined using automated blood cell counter (Mandray automated machine, model BC5300/RD28103112). Remaining blood samples were centrifuged at 3000 rpm for 15 min and plasma was stored frozen until used for biochemical assay. Measurements of plasma glucose and triglycerides (TG) were performed using standard biochemistry and the plasma viscosity was measured by simple viscometer technique, based on the rate of flow; blood viscosity was also measured.

### Statistical analysis

Results are presented as means ± standard deviation (SD). Unpaired Student’s t-test was used for continuous covariates, to compare biological parameters between the different groups. To identify risk factors associated with HTN in SCA patients, we used a binary (i.e., presence or absence of HTN) multivariate logistic model and ROC curve analysis. Significance level was defined as *p* < 0.05. Statistical analyses were done using SPSS (version 16, IBM SPSS Statistics, Chicago, IL).

## Results

The number of patients with nHTN (*n* = 65), rHTN (*n* = 29); patients with BP levels ≥ 140/90 mmHg had hypertension (HTN) (*n* = 3) [2]. However, because of the limited number of patients in the latter group, the two groups with either rHTN or HTN were pooled to create a single group (rHTN group; *n* = 29 + 3 = 32). The clinical characteristics of the study population classified according to the presence or absence of rHTN are summarized in Table 1. The rHTN group had higher weight, WC and NC when compared to those of the nHTN group. However, mean values for age, height, BMI and NC of the two groups were not statistically different (Table 1).

**Table 1:** Anthropometric data of the sickle cell anemia (SCA) patients classified according to hypertension status

Parameters	nHTN	rHTN	*P*
Age (*years*)	20.6 ± 5.5	23.6 ± 9.3	0.094
Gender (*M/F*)	30/35	20/12	
Weight (*kg*)	46.9 ± 9.1	52.5 ± 13.2	0.016
Height (*m*)	1.6 ± 0.1	1.6 ± 0.1	0.123
BMI (*kg/m*^*2*^)	19.2 ± 10.5	22.5 ± 13.9	0.202
WC (*cm*)	73.7 ± 7.1	76.9 ± 7.7	0.048
NC (*cm*)	31.9 ± 2.1	34.1 ± 1.7	0.001
Hip (*cm*)	84.6 ± 8.7	87.3 ± 10.6	0.187

Means ± SD. *nHTN* No relative systemic hypertension with blood pressure values < 120/70 mmHg; *rHTN* Relative systemic hypertension patients with blood pressure values ≥ 120/70 mmHg; *BMI* Body mass index; *WC* Waist circumference; *NC* Neck circumference. Significant difference (*p* < 0.05)

Table 2 shows the hematological and hemorheological parameters between SCA patients with rHTN and SCA patients without rHTN. Plasma viscosity was increased in the rHTN group when compared with that of the nHTN group (*p* < 0.05), whereas RBC, WBC, PLT counts as well as Hb, Hct and blood viscosity values in rHTN group were not significantly different from those of the nHTN groups (Table 2).

**Table 2:** Hematological and hemorheological profile of the sickle cell anemia (SCA) patients classified according to hypertension status

Parameters	nHTN	rHTN	*p*
RBC (*10*^*12*^*/L*)	3.2 ± 0.8	3.1 ± 0.7	0.353
WBC (*10*^*9*^*/L*)	9.3 ± 3.3	9.7 ± 4.5	0.605
Platelet (*10*^*9*^*/L*)	339.8 ± 143.0	325.4 ± 183.5	0.675
Hemoglobin (*g/L*)	48.3 ± 39.5	47.9 ± 42.8	0.966
Hematocrit (*%*)	25.5 ± 5.9	25.5 ± 5.3	0.995
Plasma viscosity	2.1 ± 0.3	2.3 ± 0.4	0.004
Blood viscosity	4.9 ± 1.8	5.3 ± 1.5	0.254

Means ± SD. *nHTN* No relative systemic hypertension with blood pressure values < 120/70 mmHg; *rHTN* Relative systemic hypertension patients with blood pressure values ≥ 120/70 mmHg; *RBC* Red blood cell count; *WBC* White blood cell count. Significant difference (*p* < 0.05)

**Fig. 1:**
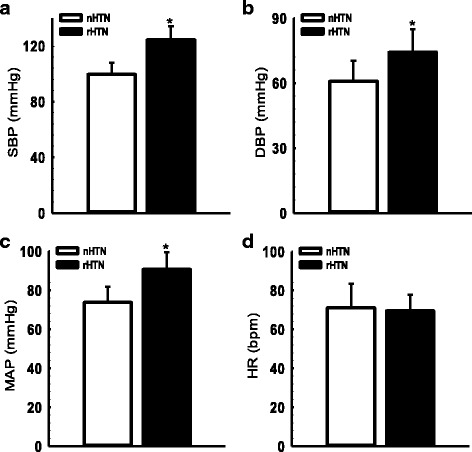
Blood pressure and heart rate of the SCA patients classified according to hypertension nHTN, no relative systemic hypertension with blood pressure values < 120/70 mmHg; rHTN, relative systemic hypertension patients with blood pressure values ≥ 120/70 mmHg; SBP, systolic blood pressure (**a**); DBP, diastolic blood pressure (**b**); MAP, mean arterial pressure (**c**); HR, heart rate (**d**). Significant difference (*p < 0.01)

Figure 1 depicts mean values for blood pressure and heart rate in rHTN and nHTN groups. Mean values of SBP, DBP and MAP were significantly higher in the rHTN group than in the nHTN group. However, no difference was observed for HR mean values between the groups.

Table 3 summarizes the mean values of CMD markers in both rHTN and nHTN groups. FPG, TG, TyG index and TyG-WC were not significantly different between the two groups. However, TyG-BMI was significantly associated with rHTN.

**Table 3:** Cardiometabolic disorder markers of the sickle cell anemia (SCA) patients classified according to hypertension

	nHTN	rHTN	*p*
FPG (*mmol/L*)	4.1 ± 1.5	4.3 ± 1.3	0.617
TG (*mmol/L*)	1.0 ± 0.5	1.1 ± 0.4	0.255
TyG index	7.9 ± 0.6	8.2 ± 0.6	0.162
TyG-BMI	151.7 ± 20.4	184.5 ± 28.0	0.044
TyG-WC	597.1 ± 70.7	638.7 ± 96.1	0.088

Means ± SD. *nHTN* No relative systemic hypertension with blood pressure values < 120/70 mmHg; *rHTN* Relative systemic hypertension patients with blood pressure values ≥120/70 mmHg; *FPG* Fasting plasma glucose; *TG* Triglycerides; *TyG index* The product of triglycerides and fasting glucose; *BMI* Body mass index; *WC* Waist circumference; TyG-BMI, TyG*BMI; TyG-WC, TyG*WC

A binary multivariate logistic model (Table 4) was used to identify risk factors associated with rHTN in SCA patients and included WC, NC, BMI, Hct, plasma viscosity, blood viscosity and TyG index. The overall model was statistically significant (chi-square = 30.38; df = 7; *p* < 0.001), however only two parameters included in the model were significantly associated with rHTN: NC (OR: 4.21; 95%CI 1.35 to 13.18, *p* = 0.013) and TyG index (OR: 7.09; 95% CI 1.38 to 36.57, *p* = 0.019). A second model, MODEL 2 adjusted for Hct, plasma viscosity and blood viscosity was still significant (chi-square = 19.44; df = 4; *p* < 0.001) and retained the same significant parameters as in MODEL 2: NC (OR: 2.36; 95%CI 1.36 to 4.11, *p* = 0.002) and TyG index (OR: 3.90; 95%CI 1.01 to 15.08, *p* = 0.048). A third model, MODEL 3 adjusted for WC was also significant (chi-square = 17.76; df = 3; *p* < 0.001). In MODEL 4, when MODEL 3 was adjusted for BMI; the overall model was statistically significant (chi-square = 19.02; df = 3; *p* < 0.001), however only NC was significantly associated with rHTN: NC (OR: 2.25; 95%CI 1.34 to 3.78, *p* = 0.002). In MODEL 5, when MODEL 3 was adjusted for both WC and BMI; the overall model was statistically significant (chi-square = 17.68; df = 2; *p* < 0.001), both NC and TyG were significantly associated with rHTN: NC (OR: 2.11; 95% CI 1.32 to 3.38, *p* < 0.002) and TyG index (OR: 4.09; 95%CI 1.10 to 15.23, *p* = 0.036). In MODEL 6, when MODEL 5 was adjusted for TyG; the overall model was statistically significant (chi-square = 12.11; df = 1; *p* < 0.001), and NC was significantly associated with rHTN: NC (OR: 1.86; 95% CI 1.22 to 2.81, *p* = 0.004); however, when MODEL 5 was adjusted for NC in MODEL 7; the overall model was not statistically significant and the association between TyG and rHTN was lost.

**Table 4:** Association between neck circumference (NC), the product of triglycerides and fasting glucose(TyG) index and relative systemic hypertension (rHTN) in sickle cell anemia (SCA) patients

NC				TyG index		
	OR	*95% CI*	*p*	OR	*95% CI*	*p*
MODEL 1	4.21	1.35–13.18	0.013	7.09	1.38–36.57	0.019
MODEL 2	2.36	1.36–4.11	0.002	3.90	1.01–15.08	0.048
MODEL 3	2.16	1.31–3.56	0.003	4.22	1.11–16.10	0.035
MODEL 4	2.25	1.34–3.78	0.002	3.69	0.98–13.85	0.053
MODEL 5	2.11	1.32–3.38	0.002	4.09	1.10–15.23	0.036
MODEL 6	1.86	1.22–2.81	0.004			
MODEL 7				2.22	0.76–6.87	0.168

To determine the relevance of each variables for predicting rHTN, the receiver operating curve (ROC) was plotted and the area under the curves (AUCs) were compared (Table 5). The AUC derived for NC was significantly larger than all other variables. For variable that was significantly associated, NC had the largest AUC (0.809), followed by weight (0.708) and Hct (0.703).

**Table 5:** Area under the receiver operating characteristic (ROC) curve in sickle cell anemia (SCA) patients with relative systemic hypertension (rHTN)

	rHTN			
	Area	SE	*95% CI*	*P*
Age	0.580	0.093	0.397–0.762	0.371
Weight	0.708	0.102	0.509–0.908	0.019
BMI	0.512	0.084	0.368–0.776	0.324
WC	0.547	0.098	0.355–0.740	0.594
NC	0.809	0.069	0.673–0.944	0.001
Hb	0.561	0.084	0.396–0.725	0.495
Hct	0.703	0.079	0.547–0.858	0.023
Plasma viscosity	0.602	0.084	0.438–0.767	0.250
Blood viscosity	0.667	0.077	0.515–0.818	0.061
TyG index	0.644	0.085	0.478–0.810	0.105
TyG-BMI	0.580	0.110	0.364–0.795	0.371
TyG-WC	0.602	0.096	0.415–0.790	0.250

*rHTN* Relative systemic hypertension patients with blood pressure values ≥120/70 mmHg; *BMI* Body mass index; *WC* Waist circumference; *NC* Neck circumference; *Hb* Hemoglobin; *Hct* Hematocrit; *TyG index* The product of triglycerides and fasting glucose; TyG-BMI, TyG*BMI; TyG-WC, TyG*WC; *SE* Standard error; *CI* Confidence interval

## Discussion

To the best of our knowledge, this is the first study that investigated the associations between increased NC and the risk of development of relative systemic elevated BP among young individuals with SCA. Multivariate logistic regression and ROC curve analyses of our data showed significant association between increased NC and the risk of rHTN among young adults with SCA. The present study demonstrates for the first time that NC is independently associated with rHTN in young adults with SCA independent of cardiovascular and metabolic risk factors.

Cardiovascular and metabolic disorders are inextricably linked and are the leading causes of mortality and morbidity in both sexes worldwide. IR is considered to be a critical metabolic link of the cardiometabolic disorder, with incidence and prevalence rapidly increasing worldwide over the last decade despite increased efforts to prevent and control. IR occurs in 20%–25% of the human population [20] and it is the hallmark of both the prediabetic state and overt type 2 diabetes [21]. Although the incidence of IR, type 2 diabetes, obesity, and CMD is considered rare in SCA; there is however a few reported cases [22, 23]. Because of the clinical as well as public health importance of IR, the ability to identify otherwise healthy normal weight non-obese individuals with IR before the development of CMD is of paramount importance, particularly in SCA patients.

The concept that some non-obese individuals present with several risk factors for CMD and represent one end of the spectrum of obesity was first proposed by Ruderman et al. ~ 30 years ago [24]. Ongoing investigations reveal that individuals that are metabolically obese but have normal weight (MONW), termed ‘metabolically abnormal normal weight’ or ‘normal weight obese’, are not uncommon [25, 26]. They are characterized by the increased levels of adiposity and IR, and a higher susceptibility to CMD [25]. Furthermore, elderly people with the MONW phenotype exhibited a higher risk of CMD and all-cause mortality [27]. In this regard, early identification of MONW individuals would have significant benefits by prompting appropriate risk detection and early management. Higher degree of IR is a representative feature of MONW, using the TyG index might help identifying SCA at risk of developing CMD.

Increased adiposity is rising to a pandemic proportion, it remains a major public health problem globally and it is an important risk factor for hypertension and CMD [11, 12]. Unfortunately, the prevalence of general and regional increased adiposity has increased in industrialized countries and also in developing countries where there is high incidence and prevalence of SCA [13]. NC is an index of upper-body fat deposit, and a reliable, simple, time saving screening measure for identification of individuals, particularly those with small stature but have excess regional body fat distribution. It has been shown to correlate positively with CMD risk factors [14, 15] such as BP and serum lipid levels [28, 29], independent of overall adiposity and central obesity [14, 16, 17]. Upper-body fat accumulation has been shown to play a pathogenic role in the development of hypertension and CMD [14–17]. It has been indicated that elevated level of free fatty acid increases oxidative stress, inflammatory and vascular endothelial dysfunction markers, which in turn play significant roles in the development of systemic hypertension [30–32]. Upper-body cutaneous fat has been shown to be responsible for the majority of systemic free fatty acids [33]. It has also been documented that increased NC is a significant predictor of obstructive sleep apnea syndrome [28], which has been associated with fluid retention-related hypertension [34, 35] and poor glycemic control, even at the earliest stages of glucose intolerance [36]. Our finding in this study is in line with evidence from other recent studies that found that measurement of NC might be helpful in early detection of prehypertension in non-obese young adult and obese children [29, 37, 38]. However, our finding was not in accordance with the observation from a recent study in Chinese adults [39]. The finding that TyG-BMI was associated with rHTN in SCA patients further corroborate the importance upper-body fat indicated by NC in this study.

Association have been reported between increased TG level and endothelial dysfunction linked vasculopathy in SCA patients [40, 41], implying that TG level could have a pathophysiological role in SCA-related complications. More so, elevated TG level has been reported to promote atherothrombotic events in patients with CMD/type 2 diabetes [42]. Our data however failed to demonstrate an association between TG level and rHTN. Although there was an association between TyG and rHTN in the binary multivariate regression model, the MODEL 3 of the binary multivariate logistic regression shows that WC has a confounding effect on the association between TyG and rHTN and that the association between TyG and rHTN was dependent on BMI (MODEL 4). We also tested whether the presence of NC could explain the association between TyG and rHTN and it was shown that the association was dependent on NC (MODEL 7). Our results therefore indicate that metabolic risk factors are likely to explain, at least in part, the development of rHTN in SCA.

In SCA patients, blood viscosity is usually lower due to low Hct and Hb [43], on the other hand, plasma viscosity is known to increase [44] than in the general population. Evidence exists that increased blood viscosity and plasma viscosity increase vascular resistance and BP [43, 44]. The results in the present study that plasma viscosity but not blood viscosity increased in the rHTN group when compared to the nHTN group may suggest that SCA patients with rHTN could have increase vascular resistance. However, failure to have significant association between rheological parameters and rHTN may rule out the possibility that rheological parameters contribute significantly to the elevated BP in this group of patients. The present study conducted in a group of SCA patients resulted in findings that might not be the unique feature of the SCA patients, but can also be applied to the general population particularly within the same age groups.

### Limitation of the study

1. Although we did not perform radiography to quantify the amount of fat accumulation in the neck directly, the stringent inclusion and exclusion criteria avoided the multiple confounding influences of medication, dietary factor, and lifestyle factor as well as assurance certainty of steady state.
2. The inability to follow up the cohort as a longitudinal study.

## Conclusion

In conclusion, our findings demonstrated for the first time that NC has a strong independent association with rHTN in SCA patients. Also, TyG index, marker of CMD could also be a useful indicator of rHTN provided NC is included. The mechanism needs to be further explored in prospective studies. Therefore, these findings clearly underscore exploring NC as a useful screening utility in early detection and stratification of systemic hypertension, particularly in individuals with SCA.

## Abbreviations

BP: Blood pressure
CMD: Cardiometabolic disorder
MONW: Metabolically obese but have normal weight
NC: Neck circumference
nHTN: No systemic hypertension
rHTN: Relative systemic hypertension
ROC: Receiver operating characteristic
SCA: Sickle cell anaemia
TyG: Triglyceride-glucose
